# Comparative Quantification of Trail-Following Behavior in Pest Ants

**DOI:** 10.3390/insects11010005

**Published:** 2019-12-19

**Authors:** Ricardo J. Vázquez, Philip G. Koehler, Roberto M. Pereira

**Affiliations:** 1 Bayer Crop Science, Waterman, IL 60556, USA; 2 Department of Entomology & Nematology, University of Florida, Gainesville, FL 32611, USA; pgk@ufl.edu

**Keywords:** tawny crazy ant, bigheaded ant, argentine ant, red imported fire ant, black crazy ant, white-footed ant, foraging behavior, pheromone

## Abstract

A comparison of trail-following movement parameters of six major urban pest ants, *Nylanderia fulva* (Forel) (Hymenoptera: Formicidae), *Pheidole megacephala*, *Linepithema humile* (Mayr), *Solenopsis invicta* Buren, *Paratrechina longicornis* (Forel), and *Technomyrmex albipes* (Smith) demonstrated several differences in velocity of movement, amplitude of the deviations from a marked trail, percent fidelity to the trail, number of curves per unit of trail, and trail-following accuracy. *Paratrechina longicornis* and *N. fulva* had the largest deviations from the marked trails and moved three times faster (25–30 mm/s) along the trail than the slowest ant, *S. invicta* (< 10 mm/s), with other ants following between these extremes. Species differences in relation to going toward or returning from food were observed in a few cases, especially with *Pa. longicornis* for which velocity, amplitude, and trail fidelity differed between the foraging and return trails. Quantification of ant trail-following movement parameters can be useful in understanding the mechanics of ant movement and may be important in testing specific strategies and products that disrupt trail-following behavior.

## 1. Introduction

Foraging behavior has been studied in several ant species such as the Argentine ant, *Linepithema humile* (Mayr) [1,2,3], the western harvester ant, *Pogonomyrmex occidentalis* (Cresson) [4], and the leaf-cutting ant *Acromyrmex octospinosus* (Reich) [5] and others. When a foraging ant leaves the nest, it follows a random or consistent path, exhibiting a higher frequency of turns further away from the nest. Once a food resource is encountered the foraging ant will communicate the quality of the food source and its location to its nestmate in an effort to recruit additional foragers [6]. Recruitment in ants includes chemical communication with other nestmates that their assistance is needed to gather resources. Ants will follow a marked trail, with movement that can be characterized by several parameters such as speed and weaving patterns. Ants use trail pheromones to mark paths to be followed by other nestmates [7], including species-specific products from various glandular sources (Dufour’s gland, hindgut, and poison gland) with varying composition and effectiveness [8]. Worker ants follow trail pheromones in a weaving (“zig-zag”) pattern [9].

Although ants may use other mechanisms to find a food source [10], ants detect trail pheromones via odor receptors in their antennae. As a worker ant follows a pheromone trail, the ant tends to move in and out of an active vapor space from one side then to the other. As one antenna, containing the odor receptors, leaves the active vapor space the ant swings back in the opposite direction exhibiting a zig-zag pattern during its time on the pheromone trail [11]. How much a certain ant deviates from a straight line as it travels between two points determines how efficient the ant movement is. Despite this general pattern, differences in the parameters that define the weaving along an established trail have not been recorded for many pest ants. The purpose of this laboratory study was to compare characteristics of foraging trails of pestiferous ants, both leaving their harborage toward a food source and returning from a food source back to their nest. Trail-following parameters may be important in understanding the efficiency of ant trail-following movement. The understanding of normal trailing following parameters but as the ant moves away from the nest, and as it moves toward the nest, it can provide important insight on the efficiency of different ant species as they forage for food. Comparison of the trailing efficiency between the paths away from the nest and the return to the nest provides information that may be useful in testing new ant control products. Also, this information is useful in testing specific strategies and products that may disrupt trail-following or decrease the efficiency of the ant trail-following movement.

## 2. Materials and Methods

Trail following patterns were compared for *Nylanderia pubens* (Forel) and *Pheidole megacephala* (Fabricius), *Solenopsis invicta* (Buren), *Paratrechina longicornis* (Forel), *Technomyrmex albipes* (Smith) and *L. humile*. Laboratory colonies were established from *N. fulva*, *S. invicta*, *P. longicornis* and *T. albipes* field collections at various sites around Gainesville, FL (Alachua County, FL, USA). Colonies of *P. megacephala* were collected in Gainesville, Dade City (Pasco County, FL, USA) and Inverness (Citrus County, FL, USA), and *L. humile* were collected from Lake City (Columbia County, FL, USA), in Florida. All laboratory ant colonies contained queens, brood and approximately 2000–4000 workers which were maintained at room temperature (22–27 °C), 64–70% RH and a photoperiod of 12:12 (L:D) h. Colonies were reared in 56 by 42 by 13 cm plastic trays with the inside walls coated with Fluon^®^ (Insect-a-slip, Bioquip Products Inc., Rancho Dominquez, CA, USA) to prevent ants from escaping. Ants were fed a regular diet of 10% sugar water, deionized water, boiled eggs, whole crickets, cockroaches, house flies, grapes and orange slices. Nest cells (150 by 15 mm Petri dish) with a plaster (Castone™ dental stone) bottom, moistened regularly, were provided for the ants to use as harborage.

Foraging arenas were clear plastic boxes (27 by 19 by 9.5 cm, Pioneer Plastics, Dixon, KY, USA) with inside walls lined with Fluon^®^. Each arena contained 400 workers, 0.10 g of brood and 1 or 2 queens per tray. Queens and brood were included in the arenas to encourage worker foraging and trail following. Previous pilot studies confirmed that queens and brood presence were necessary stimuli for the workers to actively forage and seek nutrient resources. A small nest cell (100 by 15 mm Petri dish) with a moistened plaster bottom was provided for harborage. Ants were provided no food for 24–72 h prior to trials with access to water in plastic vials (86 by 33 mm, #15 dram vials, Thornton Plastics Co, Salt Lake City, UT, USA) with a cotton stopper. For trials involving *T. albipes*, an additional Pyrex^®^ test tube (150 by 16 mm, Corning Inc., Corning, NY, USA) with a cotton stopper containing 10% sugar water was added to prevent high mortality during the starvation period. During the acclimation period, all worker ants and queens nested inside the small Petri dishes prior to experiments. Trials were conducted in a complete randomized design (CRD) with ant species as treatments and trials serving as experimental replication. Five trials were conducted with each of the six ant species.

**Figure 1:**
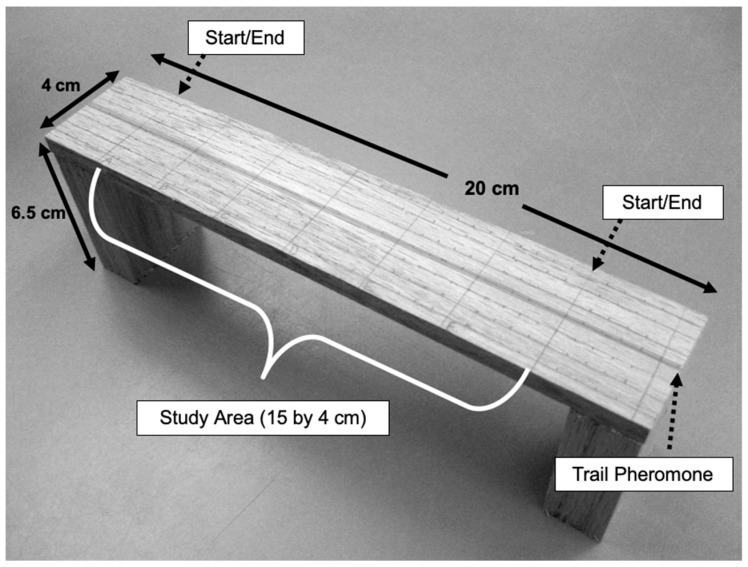
Wooden bridges (40 × 150 mm with 65 mm elevation from arena floor) used in trail-following arenas. Black lines and dotes were spaced in a 5 × 5 mm grid and served as guides for measurements of ant movement in the study area, ant path deviations away from the longitudinal pheromone trail (amplitude), and quantification of the ant movement. A gray longitudinal band was added graphically to the picture to represent the centrally applied trail pheromone.

A deli cup (242 mL, American Plastics, Chattanooga, TN, USA) with the inside and outside walls lined with Fluon^®^ was placed in the foraging arenas to serve as the site of food placement. A small slice of hard-boiled egg (~0.1 g), a slice of orange (~0.3 g) and one whole cricket (~0.8 g) were placed inside the deli cup. Ants could only access the food inside the deli cup via a wooden bridge made of red oak (20 by 4 cm by 6.5 cm high) (Figure 1). On top of the wooden bridges, a study section (15 by 4 cm) was marked with a graphite #2 pencil consisting of a 5 mm × 5 mm grid of marks, with longitudinal lines throughout the entire wooden bridge. The grid was used to determine actual position of each ant on the wooden bridge at any time. The start and endpoints of the study area were also marked on top of the wooden bridge. The bridge represented an obstacle-free, simple flat surface so that ant trail-following behavior could be studied with minimum influence from the environment, and so all ant species would be exposed to the same environment to minimize any outside influences on trail-following.

Trail pheromone extracts from each ant species were obtained by crushing the gasters of 30 workers with a glass mallet and washing in 2 mL of hexane (C6H14, Fischer Chemicals, Fair Lawn, NJ, USA). A disposable pipette was used to transfer the hexane/pheromone solution into scintillation vials. The extracts were prepared fresh when needed and refrigerated (4 °C) in a capped scintillation vial for at least 1 h prior to use. To encourage foraging in the test arenas a trail was marked on the bridge with a trail pheromone for each individual ant species. The trail pheromone was applied to the center of the wooden bridge (piles and deck) with a calligraphy pen (Koh-I-Noor® Rapidograph, Ceske Budejovice, Czech Republic) with a tip size number 0.0 (liquid line width of 0.30 mm) for a total of 0.20 mL (0.09 ant worker equivalent/cm) of trail pheromone applied on the wooden bridges. Separate bridges were used for each ant species, and any new trial, to avoid trail pheromone contamination.

A digital video camera (Sony Handycam model DCR-HC36, Sony Electronics Inc., San Diego, CA, USA) was used to record the ant trail-following patterns and a microcomputer software program for time-lapse video editing (Adobe Premier Elements 4.0, 2008) was used to process the videos and make necessary measurements. Experiments were recorded for 30 min with the digital video camera mounted 61 cm above. Ant trail-following paths (going to and returning from food source) in the videos were transposed onto graph paper.

In order to quantify the experimental parameters, the following criteria were used in establishing how ants were to be counted to avoid biases: (a) only ants starting the trail within a maximum of 5 mm deviation from the center of the bridge (pheromone trail) were counted; (b) ants were counted for 10 min maximum, or until 15 ants were counted, whichever came first; (c) the initial 10 min of videotape footage was not used in order to allow the scout ants to find the pheromone trail and recruit workers during this initial time period. For all ants that started foraging down the center trail in either direction, during the 10-min video footage examined a proportion of ants that finished the trail was calculated.

The trail-following behavior of a particular ant species was assessed by quantifying trail-following ability recorded on graph paper into the following six parameters: (a) velocity (b) amplitude, (c) fidelity, (c) number of turns, (d) trail-following accuracy, and (e) proportion of ants finishing trails. Amplitude is the measurement (in mm) of the perpendicular distance each ant species deviates from the center trail pheromone. Fidelity represents the percentage of the total bridge distance that the ant travels on top of the pheromone trail (within 1 mm of the marked trail). Turns (total number of) measures the frequency of corrective actions (left and right turns away or towards the center trail) an ant takes as it follows the trail. Velocity (mm/s) is calculated by dividing the time the ant takes to complete the 150 mm marked trail by the traveled distance. Finishing proportion measures the ratio of ants that successfully complete the trail (going to/returning from a food source) during the 10-min interval.

Trail-fallowing accuracy was calculated by the following formula:

$$TA\;=\;\left\{d\;÷\;\left[2t\;*\;\sqrt{{\left(\left[\frac{\left(d\;-\;f\right)}{2t}\right]\right)}^{2}+a^{2}}\right]\;+\;f\right\}\;\times \;100,$$

where *TA* = percent trail-following accuracy, *d* = trail distance (mm), *t* = number of turns *f* = fidelity (mm), and *a* = amplitude (mm). This formula considers that turns in the ant path increase the distance an ant has to walk between two points on the trail, and that the increase is related to the number of curves and how far the ant deviates from a straight path. The formula calculates the ant path as a series of straight lines either on the direct pheromone path or from the direct path to a point that is 1 amplitude unit away from the straight path and then back to the straight path. The length of the path that takes the ant away from, and then back to the marked pheromone trail is calculated as the hypotenuse of the right-angled triangle that has as its basis the straight line on the pheromone path, and the amplitude of the path as the height of the triangle. Given that the fidelity is the length of the path the ant stays right on the trail pheromone, the total trail distance minus the fidelity represents the portion of the ant path that deviates from the pheromone-marked trail. Thus, the total path followed by the ant can be mathematically calculated as a series of straight lines on the pheromone trials plus right-angle triangular deviations from and back to the pheromone. Since all ants were exposed to the same exact terrain on the top of the bridge, and comparisons were only made for trail-following in this uniform portion of the ant paths, direct comparison of the trails from different ants was possible. Trail-following accuracy, as presented here, is a measure of how efficient an ant species is when traveling on a straight path. Trail-following accuracy is not meant to measure the ability of an ant species to detect and follow a pheromone trail as proposed by other authors [12,13,14].

Statistical analyses were performed in JMP 13.2.0 (SAS Institute 2016). One-way ANOVA’s were used to evaluate differences in the population means with ant species as the main factor and the five parameters: amplitude, fidelity, turns, trail-following accuracy and velocity as the response variables. Treatment means were separated by Student’s *t*-test. The level of significance for all statistical analysis was set at α = 0.05.

## 3. Results

**Figure 2:**
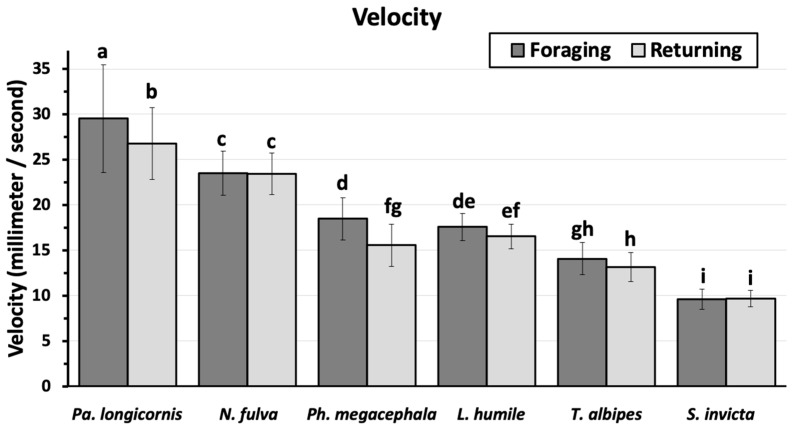
Mean ant velocity (mm/s) (mean ± 95% CI) as ants move towards a food source (Foraging) and returning to the nest (Returning). Means topped by different letters are significantly different (Students means separation; α = 0.05).

The mean velocity for the different ants varied from a maximum of 28.1 ± 0.82 mm/s for *P. longicornis* to a minimum of 9.6 ± 0.16 mm/s for *S. invicta* (Figure 2). There was no significant difference in the mean velocity between *P. megacephala* (17.0 ± 0.39 mm/s) and *L. humile* (17.0 ± 0.24 mm/s), but all other ant species moved at a different mean velocity. *Paratrechina longicornis* and *P. megacephala* workers moved faster going toward food (29.5 ± 1.35 and 18.5 ± 0.53 mm/s, respectively) than returning to the nest (26.8 ± 0.90 mm/s and 15.5 ± 0.54 mm/s, respectively). For the other ants (*N. fulva*, 23.5 ± 0.38; *L. humile*, 17.0 ± 0.24; *T. albipes*, 13.6 ± 0.27; and *S. invicta*, 9.6 ± 0.16 mm/s) there were no significant differences between the velocity of ant trail-following to and from the food source.

**Figure 3:**
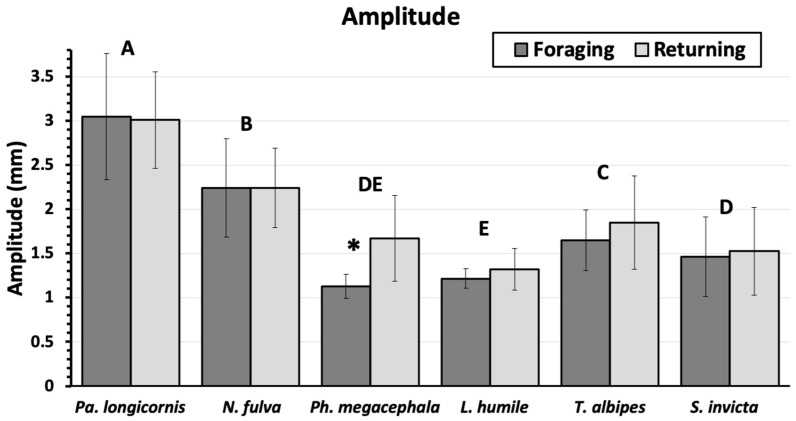
Mean trail amplitude (mm) (mean ± 95% CI) as ants move towards a food source (foraging) and returning to the nest (returning). Ant species topped by different letters are significantly different (students means separation; α = 0.05) There were no significant differences between the foraging and returning paths for any ants except for *P. megacephala* (marked with *).

There was no significant difference in mean path amplitude between the foraging and the returning paths for any ant, except for *P. megacephala* (Figure 3), which deviated more from the marked trail as the ants returned to the nest (1.7 ± 0.11 mm) than when ants went out foraging (1.1 ± 0.03 mm). However, there were significant differences among the different ant species in relation to path amplitudes (F = 82.2, *p* < 0.0001), with *P. longicornis* (3.0 ± 0.10 mm) and *N. pubens* (2.2 ± 0.08 mm) showing greater path amplitudes than *T. albipes* (1.7 ± 0.07 mm), *S. invicta* (1.5 ± 0.08 mm), *P. megacephala* (1.4 ± 0.06 mm), and *L. humile* (1.3 ± 0.03 mm).

**Figure 4:**
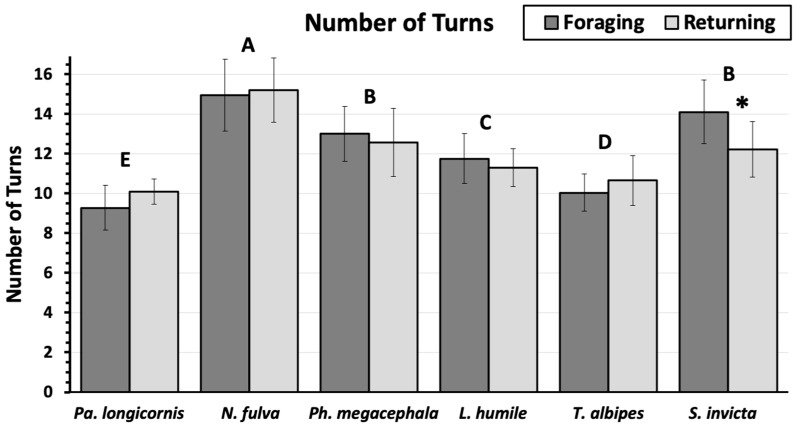
Mean total number of turns (mean ± sem) as ants move towards a food source (foraging) and returning to the nest (returning). Ant species topped by different letters are significantly different (students means separation; α = 0.05). There were no significant differences between the foraging and returning paths within the same ant species, except for *S. invicta* (marked with *).

The mean number of turns individual ants took towards and away from the center pheromone trail were significantly different (Figure 4; *F* = 82.8; df = 5; *p* = <0.0001) among *N. pubens* (15.1 ± 0.28), *L. humile* (1.5 ± 0.18), *T. albipes* (10.3 ± 0.18); and *P. longicornis* (9.69 ± 0.15), but there was no significant difference in the mean number of turns between *S. invicta* (13.2 ± 0.25) and big-headed ants (12.8 ± 0.25), although these two ants were also significantly different from all other ants. All ants had a similar number of turns both going to and coming from food, except for *S. invicta* which made significantly more turns going toward the food (14.1 ± 0.37) than going back to the nest (12.2 ± 0.32).

**Figure 5:**
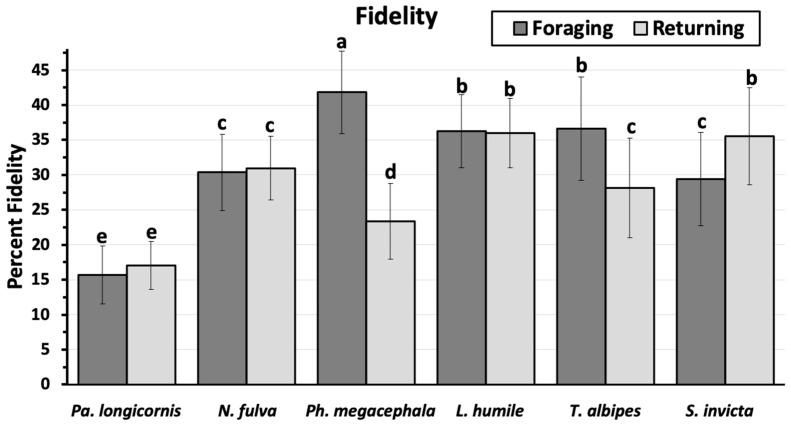
Mean percent trail fidelity (mean ± sem) as ants move towards a food source (foraging) and returning to the nest (returning). Means topped by different letters are significantly different (students means separation; α = 0.05). Significant differences between the foraging and returning paths were detected for *P. megacephala, T. albipes*, and *S. invicta*.

The mean percent fidelity did not vary between the paths going toward the food and going back to the nest for *P. longicornis* (16.4 ± 0.62), *N. fulva* (30.7 ± 0.81), and *L. humile* (36.1 ± 0.82) (Figure 5). However, both *P. megacephala* and *T. albipes* had greater trail fidelity going toward the food than returning to the nest, while *S. invicta* had the opposite behavior, having greater trail fidelity returning to the nest.

**Figure 6:**
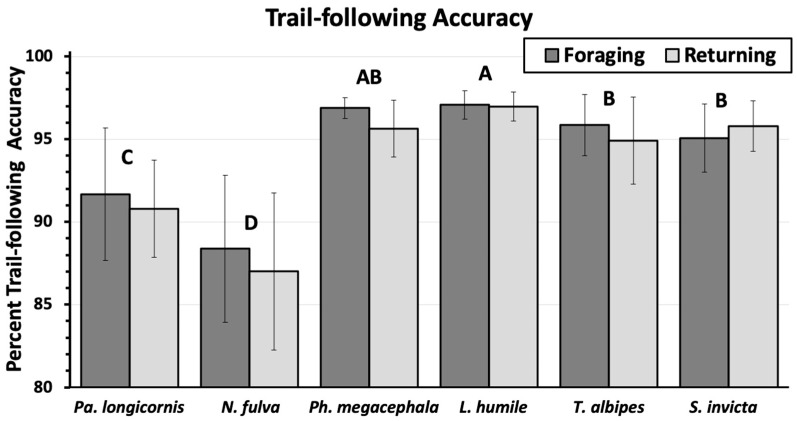
Mean trail-following accuracy (mean ± sem) as ants move towards a food source (Foraging) and returning to the nest (Returning). Ant species topped by different letters are significantly different (Students’s means separation; α = 0.05). There were no significant differences between the foraging and returning paths within the same ant species.

There as a significant difference (F = 68.2, *p* < 0.0001) in the trail-following accuracy which basically put the ants in two groups, with the crazy ants having lower trail-following accuracy (86–92%) and the other ants with higher accuracy (94–98%) (Figure 6). There were no significant differences in accuracy (F = 3.19, *p* = 0.07) between the track toward the food and the track back to the nest for any of the ants, although the general trend for most of the ants was a slightly lower trail-following accuracy for the return track. However, we detected no differences in the proportion of ants finishing trails, for any of the tested species, either going to or returning from the food.

Despite the different trail parameters being used in the calculation of the trail-following accuracy, the only parameter with high correlation (r^2^ = 0.400–0.751) with the trail-following accuracy was the amplitude. Greater amplitude led to lower trail-following accuracy, whereas lower amplitude corresponded well with higher trail-following accuracy. No statistically significant correlation was observed between the other trail parameters measured in this study.

There was no significant difference among the ant species for the proportion of starting ants that actually completed the trail, (F = 0.29, *p* = 0.92) or between ants going away from the nest of returning to the nest (F = 0.44, *p* = 0.51), with an 81.8 ± 10.41% completion rate for ants moving away from the nest and a 79.6 ± 11.94% completion rate for ants coming back to the nest.

## 4. Discussions

Clear behavioral differences in trail-following patterns are demonstrated here for six major pest ant species, with an obvious separation between the crazy ants, *P. longicornis* and *N. fulva*, and the other pest ant species in several aspects of their trail-following behavior. The most striking difference in the measured parameters is on the trail-following speed, with the crazy ants showing a 2 to 3 times faster pace than other ants, but also with greater amplitude and lower trail fidelity than the other tested ants, resulting in a significantly lower trail-following accuracy for the crazy ants. A behavioral adaptation to moving fairly quickly and more erratically outside of the nest has the advantage of preventing exposure to both the elements and predation. Conversely, the very aggressive fire ant had the lowest trailing velocity, which may be related to its high degree of aggressivity [15].

Potentially, it can be advantageous in maximizing food discovery. However, despite all the differences observed in the trail parameters of the study ants, the ability of the ants to complete the trail was fairly consistent among the different species. This indicates that the different trail-following strategies may be more a result of physical characteristics of the ants and less a consequence of an evolutionary process affecting the trail-following behavior.

There were some interesting discrepancies between the different parameters measured or calculated for the foraging behavior of the study ants. For instance, despite *N. fulva* staying on the marked trail approximately twice as long as *P. longicornis*, trail-following accuracy for the tawny crazy ants was significantly lower than that for the black crazy ant. The tawny crazy ant seems to follow the marked trail fairly well, but when it deviates from the marked trail, those deviations either take the ants farther from the marked trails or for longer periods. Comparatively, the other ants in the study had fairly high fidelity to the marked trail, which, combined with a low amplitude of the deviations, resulted in high trail-following accuracy for *P. megacephala, L. humile, T. albipes* and *S. invicta*.

Because ant trails are normally bidirectional [16], and, in our experiments, ants were encouraged to maintain that aspect because of a single marked path on the wooden bridge, some of the movements away from the marked path were dictated by the presence of other ants traveling in the opposite direction. Differences in the fidelity between ants leaving the nest and ants returning to the nest are a consequence of this bidirectional movement.

The ants’ fidelity to the trail fall into three different patterns in the study ants: (a) ants with similar fidelity whether moving away or toward the nest, represented by *P. longicornis*, *N. fulva*, and *L. humile*; b) ants with greater fidelity to the trail when moving away from the nest, represented by *P. megacephala* and *T. albipes*; and (c) ants with greater fidelity on the return trip to the nest, represented by *S. invicta*. Greater fidelity to the trail on the return trip to the nest would seem to be the preferred behavior, assuming that an ant laden with food should move more directly to the nest, both the minimize risks and to save energy. This is especially true when ants are exposed to risks present in the field that were absent in our studies. However, low trail fidelity can be advantageous to ants [17].

Despite our measurements being taken in a very uniform wooden path with a well-marked path for the ants to follow, trail-following accuracy showed great variation between the group of the crazy ants with trail-following accuracy at 91% and below, and the other ants in the study, which despite considerable taxonomic and ecological differences, had fairly uniform trail-following accuracy between 95% and 97%. Trail accuracy seems to be the best measurement of the erratic behavior of the crazy ants, which can be detected even when the ants are following precise trails and not responding to outside stimulus as is the case when ants are disturbed during foraging.

In this work, the trail-following patterns are quantitatively described for the six major pest ant species using parameters that quantified the zig-zag trail-following pattern in ants. Understanding trail-following behaviors of invasive pest ants is an important factor that may allow a greater understanding of why these ants become efficient pestiferous species. Also, a better understanding of foraging behaviors of pest ants may play a role in the development and refinement of control strategies that best suit different pest ant species, such as toxic bait placement around the established trail, and application of products that may disrupt trail-following behavior.

## 5. Conclusions

Trail-following patterns for major pest ant species vary in relation to velocity, amplitude, fidelity, number of turns, and trail-following accuracy, falling into three different patterns with (a) similar trail fidelity whether moving away or toward the nest; (b) greater fidelity to the trail when moving away from the nest; and (c) greater fidelity on the return trip to the nest. Trail-following behavior is an important factor in understanding the efficiency of pest ant species.
